# Retraction Note: Downregulated poly-C binding protein-1 is a novel predictor associated with poor prognosis in acute myeloid leukemia

**DOI:** 10.1186/s13000-018-0745-5

**Published:** 2018-09-12

**Authors:** Meifeng Zhou, Xiuzhen Tong

**Affiliations:** Department of Hematology, the First Affiliated Hospital of Sun Yat-sen University, Guangzhou, 510080 Guangdong China

## Retraction Note: Zhou and Tong. BMC Diagnostic Pathology (2015) 10:147 DOI 10.1186/s13000-015-0377-y

This article [1] is retracted at request of the Editor. After publication of this article [1] concerns were raised regarding inadequate documentation of the statistical methodology and use of inappropriate statistical analyses in the study. For these reasons the conclusions of the study cannot be considered to be supported by the data. The authors do not agree to this retraction.
